# In Search of Consistent Predictors of Children’s Physical Activity

**DOI:** 10.3390/ijerph14101258

**Published:** 2017-10-20

**Authors:** Keren Best, Kylie Ball, Dorota Zarnowiecki, Rebecca Stanley, James Dollman

**Affiliations:** 1 Institute for Physical Activity and Nutrition, Deakin University, Burwood, VIC 3125, Australia; kylie.ball@deakin.edu.au; 2 School of Pharmacy and Medical Sciences, University of South Australia, Adelaide, SA 5001, Australia; dorota.zarnowiecki@unisa.edu.au; 3 Early Start Research Institute, University of Wollongong, Wollongong, NSW 2500, Australia; rstanley@uow.edu.au; 4 School of Health Sciences, University of South Australia, Adelaide, SA 5001, Australia; james.dollman@unisa.edu.au

**Keywords:** children, adolescents, physical activity, sport, predictor, correlates, social ecological model, health promotion

## Abstract

Physical activity is pivotal for children’s health and well-being, yet participation declines across teenage years. Efforts to increase physical activity need to be strengthened to combat this, however, evidence for the design and planning of physical activity promotion in children is lacking. The aim was to identify predictors of physical activity that were relatively consistent across three different measures of physical activity, in pre- and early adolescent South Australians. This is the first study to compare correlates of physical activity across three measures of physical activity in a single sample, in this age group. Children (*n* = 324) aged 9–13 years and their parents were surveyed on personal, interpersonal and environmental correlates of physical activity. Child physical activity was objectively measured using pedometers (7 days). Self-reported physical activity was determined from organised sport participation and the Physical Activity Questionnaire for Adolescents. Regression models were used to identify consistent predictors of three physical activity measures. Consistent predictors across multiple physical activity measures were: parent support for physical activity, having appropriate clothing for sport, enjoyment of physical activity and perceived availability of sporting clubs. These predictors identify potential avenues for directing intervention efforts to increase physical activity in early adolescents.

## 1. Introduction

The cardiovascular, skeletal, psychological and social benefits of regular physical activity for children and adolescents are supported by an abundant and growing literature [1]. Data on secular trends for this age group, however, suggest that physical activity promotion efforts have met with limited success [2]. Further, there is a precipitous decline in physical activity engagement across the teenage years [3], highlighting the urgent need to understand influences on physical activity among young people. This is of further concern as physical activity and inactivity behaviours among children and adolescents tend to track into adulthood [4].

The pre-adolescent years represent a critical period for the promotion of free, unstructured play as well as participation in organised, structured activities such as sport and physical education [5]. There is prolific literature describing correlates of physical activity behaviours in young people. Correlates have most often been studied according to a social ecological framework that acknowledges the complex interrelationships among personal, interpersonal, environmental and policy domains of influence [6]. Despite widespread agreement on the appropriateness of an ecological basis for study design, the weak links between our understanding of causation and physical activity promotion cast doubt on the utility of the evidence at hand. Heterogeneity in the design of correlates research has hampered understanding of those factors that are most consistently predictive of physical activity in young people, with studies differing according to sample characteristics, measurement instrumentation and the context of physical activity under examination [7]. Correlates of physical activity have been shown to vary according to gender [8,9], age [2], and socioeconomic status [10,11] of study samples. Correlates are also shown to differ according to the approach to physical activity assessment, such as objective measures and self-report [12], and physical activity context, such as free play and organised sport [5,13]. Overall, the current literature still presents a confusing evidence base for intervention and policy design, underscoring the need for systematic data gathering and analysis.

Over the last 20 years, physical activity research has seen the emergence of objective measures, such as pedometers and accelerometers, to complement self-report approaches such as diaries and recalls. These objective instruments can provide relatively accurate data on overall physical activity volume and intensity and have therefore advanced our understanding of the associations of physical activity and health outcomes. Nevertheless, self-report approaches, while prone to recall biases [14], are required for important contextual information, such as how, where and with whom physical activity is performed, that informs promotion planning. Accordingly, it is acknowledged that there is no single best measure of physical activity [15,16].

The current study simultaneously tested associations of correlates, hypothesised from a social ecological framework and self-determination theory [17], and physical activity assessed by multiple means in a sample of sociodemographically diverse pre- and early adolescent Australians, stratified by sex.

The aim of the study was to identify predictors of physical activity that were relatively consistent across physical activity outcomes with a view to providing a robust evidence base for policy and initiatives that more effectively slow the worrying decline in physical activity participation accompanying adolescence.

## 2. Materials and Methods

### 2.1. Participants and Recruitment

Data for this study were collected as a part of the ‘Resilience in Eating and Activity for Child Health’ (REACH) study, a cross-sectional investigation of the predictors of 9 to 13-year-old children’s eating and physical activity behaviours [18,19]. The REACH study was conducted in Adelaide, South Australia from February to November 2010. Data collection relevant to this analysis involved two stages; children’s questionnaires that were computer-administered in schools, and a computer-assisted telephone interview (CATI) with a subsample of parents. Ethics approval was received from the University of South Australia Human Research Ethics Committee (Protocol number: P278/08) and the South Australian Department of Education and Children’s Services Ethics Committee (Protocol number CS/09/0922.2), and participants gave informed consent before participating.

Participants were recruited from grades five, six and seven in Government primary schools in the Adelaide metropolitan area, using a purposive recruitment method to capture a diversity of socioeconomic position (SEP). All eligible schools were first stratified by SEP using the School Card Register (SCR), a school ranking representing the proportion of students receiving means-tested Government assistance to meet the cost of school attendance. All metropolitan Government primary schools were classified into low, mid and high SEP tertiles using the most current SCR rankings (2007–2008), and schools were randomly selected from each tertile using a random number generator. Rolling recruitment was conducted concurrent with the first stage of data collection, allowing for the number of participants from each socioeconomic tertile to be monitored and recruitment to be adjusted to attain an even distribution. Eighty two schools were approached and 27 schools (32.9%) agreed to participate. In total, 2575 children received information about the REACH study, and written consent was received from 1257 parents (48.8% response rate). The physical activity questionnaire was completed by 1204 children using school computers and assisted by one research assistant per 10 children, and 410 parents completed the CATI. Compliant pedometer data (described below) were provided by 846 children.

### 2.2. Study Measures

#### 2.2.1. Socioeconomic Variables

Parents reported socioeconomic information about their families in the CATI. Mother’s and father’s education levels were reported on an eight-point scale ranging from (1) never attended school, to (8) completed postgraduate education. Annual gross household income, including pensions and government assistance, was reported using seven income brackets ranging from (1) up to AU$12,000, to (7) more than AU$100,000. The income was adjusted by the number of individuals in the household who were dependent on that income, to form the variable ‘equivalised income’. Residential postcode was used to derive an area-level indicator of SEP based on the Socio-economic Index for Advantage (SEIFA) [20].

#### 2.2.2. Objective Measurements of Physical Activity: Pedometry

Children wore a New Lifestyles (NL) 1000 pedometer for seven consecutive days. Pedometers were worn at the right hip, in line with mid-axilla, clipped to clothing. Previous studies have found the NL1000 to have excellent validity and reliability [21,22,23]. Pedometer records with fewer than 1000 steps day^−1^ on any day were excluded (*n* = 14) [24], as were days on which the pedometer was removed for a total of more than four hours during waking hours, as recorded on a log sheet (*n* = 237). Average daily steps (‘Daily steps’) was calculated when at least 3 weekdays and one weekend day met compliance criteria.

#### 2.2.3. Self-Reported Physical Activity: Physical Activity Questionnaire for Adolescents; PAQ-A

The PAQ-A asks respondents to recall the number of times in the last week they performed moderate to vigorous physical activity, choosing from a checklist. Seven questions assess physical activity in both in-school and out-of-school-hours, covering physical education, lunch, after school, evenings and the weekend. A composite index is calculated from the average of seven items to reflect the overall physical activity level (PAQ score). The PAQ-A has been used in many countries and has acceptable validity and reliability in the age range of the current study [25,26].

#### 2.2.4. Self-Reported Physical Activity: Organised Sport Participation

A question was added to the PAQ-A that asked children to list the organised club and school sports played in the previous 12 months (‘Sport’).

#### 2.2.5. Child-Reported Predictors of Physical Activity

Items in the children’s questionnaire used to derive personal and interpersonal predictor variables were adapted from the eat well be active Community Programs (*ewba*) conducted in metropolitan and rural South Australia between 2006 and 2009 [27].

##### Personal Variables

Children reported their date of birth at the time of data collection and decimalised age was calculated. ‘Screen time’ was represented with a single item; ‘On a typical school day, how many total hours outside of school do you watch TV, view videos or work/play on the computer?’. Six response options ranged from (1) none to (6) more than 4 h.

For all of the personal variables that follow, the response options were: strongly agree; agree; unsure; disagree; strongly disagree. Perceived outcomes of regular physical activity were assessed with multiple items, beginning with ‘Playing games or sports over the next year might help me...’ and ending with: ‘keep me healthy’; ‘get me fit or help me stay fit’; ‘study and learn better’; ‘have lots of fun’; ‘make my parents/carers happy’; ‘spend time with my friends’; and ‘make new friends’. An ‘Outcome expectancies’ factor was derived as the average of individual outcomes, with a Cronbach alpha of 0.81.

Barriers self-efficacy was assessed from items beginning with ‘I could still play sport or games even if…’ and ending with: ‘others made fun of me’; ‘there is no-one to do it with’; ‘I was not good at it’; ‘I had no help to get to training and games’; ‘my parents/carers did not encourage me’; and ‘my friends did not take part’. A ‘Barriers self-efficacy’ factor was derived as the average of individual outcomes, with a Cronbach alpha of 0.78.

Single items represented: enjoyment of physical activity (‘Like PA’), ‘I like playing sports and games’; perceived competency (‘Good at PA’), ‘I think I am good at sports and games’; and personal barriers (‘Don’t like PA feel’), ‘I don’t like how being active makes me feel (e.g., hot, sweaty, out of breath)’.

##### Interpersonal Variables

For all of the social variables that follow, the response options were: strongly agree; agree; unsure; disagree; strongly disagree.

‘Parent support’ was calculated as the average of four items, with a Cronbach alpha of 0.71; ‘How often does your father/male carer (or mother/female carer) help you to play some sort of sport or physical activity, for instance take you to sport or give you money for sport?’; and ‘How often does your father/male carer (or mother/female carer) encourage you to play some sort of sport or physical activity?’.

The following items on parent influence were entered into models separately, as the Cronbach alpha was substantially reduced when added to the previous two parent support items: ‘How often does your father/male carer (or mother/female carer) play some sort of sport or physical activity with you?’ (‘Parent play with’); and ‘I have the right clothes or shoes for sport’ (‘Clothing’).

Rules imposed by parents were represented by ‘My parents/carers let me watch as much TV as I like at home’ (‘Rules’).

Positive influence of peers was represented by the following items: ‘How often does your best friend or their family encourage you to play some sort of sport or physical activity?’ (‘Friend encourage’); and ‘How often does your best friend play some sort of sport or physical activity with you?’ (‘Friend play with’).

Barriers associated with peers were represented by the following single items: ‘Other kids make fun of me when I am physically active’ (‘Other kids tease’); and ‘It is not safe to play at school because of bullies’ (‘Bullies’).

##### Environmental Variables

The only physical environment variable in the child questionnaire was ‘There are lots of clubs near where I live that I can join to play sport’ (‘Clubs’).

#### 2.2.6. Parent-Reported Predictors of Physical Activity

CATIs were conducted with parents after the school visit phase of data collection was completed. If a parent was unable to participate in the interview at the time of contact, a more suitable time was arranged. If participants were not reached, up to 10 attempts to contact them were made.

Environmental and interpersonal variables included in the CATI were adapted from items used in the *ewba* parent questionnaire [27] and the SocioEconomic Status and Activity in Women (SESAW) study physical activity questionnaire [28,29].

##### Interpersonal Variables

Social capital was represented by a single item; ‘How many of your neighbours do you know by name?’ (‘Friendly neighbours’) [30].

##### Environmental Variables

For all safety variables, the response options were: strongly agree; agree; unsure; disagree; strongly disagree. Neighbourhood safety (‘Safety’) was calculated as the average of responses to the following items, with a Cronbach alpha of 0.66: ‘Your closest park/playground from home is safe for your child to play in’; ‘It is safe for your child to walk or ride a bike alone in your neighbourhood during the day’; ’There are busy roads to cross when out walking or bike riding in your neighbourhood’; ‘Dogs frighten people who walk or bike ride in your neighbourhood’; ‘There is a lot of traffic in your neighbourhood’; ‘You are worried about older kids hanging around the neighbourhood’; and ‘You are worried about strangers in the neighbourhood’.

Neighbourhood walkability (‘Walkability’) was represented as the average of two items, with a Cronbach alpha of 0.65: ‘Your neighbourhood is well-maintained and attractive’; and ‘There are footpaths on most of the streets in your neighbourhood’. Response options were: strongly agree; agree; unsure; disagree; strongly disagree.

Costs associated with physical activity participation (‘Cost’) have been included as an environmental variable as this relates to the policies and practices of external providers such as clubs and schools. The following three items were averaged, with a Cronbach alpha of 0.72: ‘The cost of purchasing sports equipment limits how much equipment you buy for your child, for example bikes, nets, bats and balls’; ‘The cost of club sport limits my child’s participation in club sport, for example, costs associated with uniform and registration fees’; and ‘The cost of school sport limits my child’s participation in school sport’. Response options were: strongly agree; agree; unsure; disagree; strongly disagree.

Venues in the neighbourhood for physical activity were represented by individual items (yes/no response options) that were entered into models separately: ‘Is there a park or playground within walking distance from your home where your child can play? (‘Playgrounds’); and ‘Are there sporting facilities in your neighbourhood for your child to use, for example ovals, courts, skate ramp or recreation centre?’ (‘Facilities’).

### 2.3. Data Analysis

Means and standard deviations were calculated for outcome and predictor variables, and independent *t* tests were performed to compare boys and girls on all variables (see Table 1). Product moment correlations were calculated between all physical activity variables and potential correlates (see Table 2). Correlations among predictors were assessed for multicollinearity; as coefficients were >0.40 between ‘Like PA’ and ‘Good at PA’ among both boys (*r* = 0.52) and girls (*r* = 0.61), these variables were entered separately into regression models.

Regression models for personal, interpersonal and environmental variables were built separately for each physical activity outcome (Daily steps, PAQ score and Sport). Predictors that were correlated with a physical activity variable (*r* > 0.10; see Table 2) were then entered into the regression model for that variable. Linear models were constructed for Daily steps and PAQ score, and logistic models for Sport (Yes/No), stratified by sex, with robust standard errors accounting for clustering of variables in schools. Forward stepwise entry was used to identify those variables that were independent predictors of the dependent physical activity variable. Sociodemographic variables (parent education, equivalised income and SEIFA score) were forced into each model at the first step. Analyses were performed using Stata (version 14; StataCorp LP, TX, USA) with significance inferred if *p* < 0.05.

## 3. Results

### 3.1. Comparisons by Sex

Boys were more physically active than girls according to the ‘global’ measures of Daily steps and PAQ score, but not for Sport (see Table 1). Among predictor variables, higher scores were evident among boys for the following personal attributes: Outcomes expectancies (*p* = 0.001), Barriers self-efficacy (*p* = 0.03), ‘Like PA’ (*p* < 0.0001) and ‘Good at PA’ (*p* < 0.0001). Girls were more likely to report that they did not like how physical activity made them feel; ‘Don’t like PA feel’ (*p* < 0.0001). There were no differences between boys and girls for any of the interpersonal and environmental predictor variables.

### 3.2. Associations of Demographic, Personal, Interpersonal and Environmental Variables with Physical Activity Variables

Table 2 displays the bivariate associations of predictor and physical activity variables. Parent education was unrelated to individual physical activity variables, while equivalised income (girls only) and SEIFA score (boys and girls) were positively associated with Sport. Child personal factors were consistently associated with physical activity, regardless of sex and the physical activity measure. The personal predictor variables Outcome expectancies, Barriers self-efficacy, Like PA and Good at PA were more strongly correlated with the self-reported physical activity variables (PAQ score and Sport) than with Daily steps. Among interpersonal factors, Parent support and ‘Parents play with’ were largely associated with self-reported physical activity variables, while Parent support was associated with Daily steps among girls only. Having the appropriate clothing for physical activity was positively associated with most physical activity variables. Peers influenced children’s physical activity most consistently through encouragement (‘Friend encourage’) and positive modelling (‘Friend play with’). Negative peer influences through teasing (‘Other kids tease’) and bullying (‘Bullies’) showed scattered and relatively weak associations with physical activity. Among environmental variables, Clubs was positively associated with self-reported physical activity variables while correlations with Safety and Walkability were less consistent. Cost and Playgrounds were largely unassociated with physical activity variables.

### 3.3. Regression Models

Table 3 (boys) and Table 4 (girls) display the significant variables from bivariate associations (*p* ≤ 0.10) that were retained after multivariable forward stepwise regression analyses. Considering the model of personal variables, ‘Like PA’ was the only variable associated with more than one physical activity variable (PAQ score and Sport) among boys. Among girls, the personal attributes associated with more than one physical activity outcome were Barriers self-efficacy, ‘Good at PA’ and ‘Like PA’.

From boys’ interpersonal models, Parent support and Clothing each predicted two physical activity variables. Peer support (‘Friend encourage’ and ‘Friend play with’) were independent predictors of PAQ score only. Among girls, Parent support (3 physical activity variables) and Clothing (2) were consistently associated with physical activity variables.

From environmental models, Clubs was retained in two models (PAQ score and Sport) among boys, while no environmental variables predicted more than one physical activity variable in girls.

## 4. Discussion

This study identified consistent predictors of children’s physical activity across all levels of the social ecological framework. The data also revealed sex-specific predictors that should be considered when developing physical activity promotion strategies for 9–13 year olds.

Consistent with existing evidence, boys were more active than girls [31,32,33]. However, the factors responsible for these differences are still not well understood. In this study, it was shown that there were no differences between boys and girls for any of the interpersonal and environmental predictor variables but personal factors were found to be predictors of physical activity. This is not surprising as the social ecological theory [6,34] posits that influences most proximal to the target group under investigation tend to have a greater impact on physical activity behaviour [6,35]. In addition, girls reported less favourable personal attributes compared to boys, including lower enjoyment (‘Like physical activity’), perceived physical competency (‘Good at physical activity’) and lower perceived ability to overcome barriers (Barriers self-efficacy). These variables were consistent predictors of both objectively assessed and self-reported physical activity in girls. The consistency of these findings across different measures of physical activity suggests that these predictors are salient for girls’ physical activity and should be a target for interventions and programs. These findings are consistent with other studies that also show personal variables as strong predictors of girls’ physical activity [9,36,37].

Psychological variables relating to enjoyment, and confidence to overcome barriers and in skill ability are likely to be limiting factors for physical activity engagement in girls rather than boys [38]. There are a number of potential explanations for these gender differences. Previous research suggests that socio-cultural factors may be at play where males tend to be socialised to have a stronger affinity to more strenuous and competitive activities compared to girls, who tend to gravitate to more cooperative, less vigorous activities [37,39]. This is also supported in the school-based literature, which has shown that school-based activities are often set up to be masculine-oriented [40]. Engagement in physical activity, particularly activity levels gained through physical games and sports, is reliant on a certain level of skill competency. Attraction and motivation to engage in these physical activity experiences is contingent on children’s perceptions of competency. In the present study, girls reported more negative perceptions of physical activity competency, which may influence their enjoyment, motivation to engage and their development of physical competency [38]. These fundamental differences require specific consideration in the design of physical activity programs and interventions for girls. With girls being at higher risk of insufficient levels of physical activity, consideration needs to be given to activity options that are compatible with girls’ preferences and expectations [37]. This is of vital importance as research shows that children who enjoy physical activity and are self-determined tend to transfer this across multiple contexts [41]. By addressing these modifiable predictors during childhood, there is greater chance of reducing these gender differences into adolescence.

Enjoyment of physical activity (‘Like PA’) was a consistent predictor of physical activity outcome variables in both sexes. This finding is consistent with other studies that have reported child enjoyment as a motivator of participation in physical activity [42,43,44]. For children, physical activity is about hedonistic enjoyment rather than any long term investment in health [45]. As children tend to engage in activities they enjoy [45], providing a variety of physical activity opportunities to engage in, to identify preferences, could help to increase physical activity levels in this population.

Parent support was a consistent predictor of different indicators of physical activity in boys and girls. This finding is in accord with other literature, including a recent study of USA children of a similar age range, in which family support predicted physical activity represented by PAQ score and moderate- to vigorous-intensity physical activity from accelerometry, in both boys and girls [12]. Wenthe and colleagues [12] noted that females reported lower perceived family support than boys, in contrast with the current study which found no gender differences in perceived parent support. Possibly, parents in the current study were relatively ‘even-handed’ in their roles as facilitators of their children’s physical activity and were unaffected by the stereotypically gendered perspectives on children’s physical activity needs and preferences.

Having the appropriate clothing was consistently associated with physical activity variables in boys and girls, and may reflect the instrumental role that parents play through meeting costs associated with participation. The cross-sectional study design masks causal pathways; it could be argued that clothing is an outcome rather than a determinant of physical activity, given that a child taking part in sport will be provided with the appropriate clothing. Nevertheless, parents act as gatekeepers to children’s physical activities in a range of interrelated ways that resonate with the central constructs of self-determination theory [17]: autonomy, competency and relatedness. These include: instrumental and direct support to engage in a wide range of activity choices; emotional and motivational support such as providing encouragement and praise; and observational support such as role modelling of behaviours [46]. While the current study represented parent support only by encouragement and sharing activities with children, the findings add to the evidence that parents play a crucial role in a child’s physical activity levels. The robustness of parent support as a predictor in the current study should prompt deeper research of the relative importance of specific sources of parental influence in order to refine interventions with a family focus.

Neighbourhood safety was unrelated to physical activity outcomes. In all, the literature is inconsistent on the relationship between neighbourhood safety and children’s physical activity, regardless of whether safety is represented as parents’ perceptions or actual statistics such as crime rates and traffic density [47,48]. The mixed and generally weak associations reported in the literature may be attributable to the relatively high and invariant ratings of safety reported by survey respondents, which would then limit the detection of associations in statistical models, but should not be interpreted as a lack of relevance to physical activity promotion among young people.

The perceived availability of clubs predicted physical activity in both sexes, for boys across PAQ score and Sport, and for PAQ score in girls. Physical activity occurs in both organised and non-organised contexts, and opportunities for organised participation are important, perhaps more so with the decline in school sport opportunities observed in some demographic sub-groups of children [49]. A recent study found that self-reported participation in organised sports was associated with more time spent in accelerometer-assessed physical activity in children and adolescents [50]. National Australian data show that organised sport participation rates decline during adolescence [51], highlighting the importance of sports club engagement as an avenue for increasing physical activity in young people and therefore the need for ongoing funding to support this.

This study has several strengths. Generalisability of findings was enhanced in that the sampling methodology ensured a wide diversity in SEP and achieved a relatively large sample. The parent and child questionnaires were thoroughly pilot tested in appropriate samples. Computer administration and using the sophisticated CATI ensured quality data collection and maximised responses from children and parents, respectively. There was a relatively high ratio of research assistants to students to assist children with low reading and cognitive skills, improving confidence in data. The physical activity predictors explored in this study represent multiple levels of the social ecological framework.

This study is one of only a few investigating a broad range of potential influences on children’s physical activity that persist across multiple physical activity measures. It is important, however, to acknowledge a number of limitations. Cross-sectional design prohibits inferences about temporal relationships, and cannot shed light on reciprocal associations. Further, predictors of change may differ from predictors of current behaviours. Predominantly mothers (87%) participated in the CATI, and perceptions of home and neighbourhood may differ in important ways between male and female caregivers, and in single parent homes. While the data was collected 7 years ago, comparisons to current literature are still relevant. The study employed a one-dimensional measure of sport (any organised sport undertaken in the previous 12 months) which does not capture the frequency, duration and intensity of participation, including training. Weight status was not measured due to time limitations and sensitivities in the state education system at the time of the survey. It was therefore not possible to test interactions of predictors of physical activity and weight status. A reliability study was not conducted for the child questionnaire, due to the already considerable burden on school time and resources.

## 5. Conclusions

Assessing predictors of physical activity, nested in an ecological framework, across a range of both objective and subjective measure is a unique approach to identifying those factors that are consistent influencers of children’s physical activity, irrespective of the measurement approach. This study identified several predictors of children’ physical activity that are consistent across different measures and should, therefore, be prioritised and targeted in physical activity promotion. Parent support for physical activity, having appropriate clothing for sport, enjoyment of physical activity and perceived availability of sporting clubs were all associated with child physical activity, across multiple physical activity measures. Our findings suggest that intervention designers as well as education or junior sport policy makers should incorporate components and targeted strategies that focus on these modifiable factors.

While this study presented some important avenues for directing intervention efforts to increase physical activity in early adolescents, further research into other predictors and the underlying mechanisms of how these factors influence children’s physical activity is necessary for maximising the impact of physical activity promotion.

## Figures and Tables

**Table 1:** Descriptive statistics for outcome and predictor variables.

Measured Variables	Boys	Girls	*p*
**Physical activity**			
Daily steps	12,490 (2949)	10,112 (2397)	<0.0001
PAQ score	3.29 (0.70)	2.97 (0.66)	<0.0001
Sport			
Number of sports	1.65 (1.32)	1.55 (1.27)	0.15
No sports (%)	16.7	17.2	0.44
**Personal**			
Age (years)	11.35 (0.93)	11.25 (0.92)	0.33
Outcome expectancies	4.31 (0.66)	4.18 (0.62)	0.001
Barriers self-efficacy	4.20 (0.70)	4.11 (0.66)	0.03
Like PA	4.69 (0.67)	4.47 (0.76)	<0.0001
Good at PA	4.23 (0.84)	3.90 (0.90)	<0.0001
Don’t like PA feel #	3.46 (1.30)	3.14 (1.13)	<0.0001
**Interpersonal**			
Parent support	3.38 (0.97)	3.47 (0.89)	0.37
Parent play with	2.65 (0.91)	2.68 (0.94)	0.77
Clothing	4.31 (0.87)	4.24 (0.86)	0.48
Rules	3.22 (1.19)	3.20 (1.08)	0.91
Bullies #	4.26 (0.88)	4.24 (0.88)	0.81
Other kids tease #	4.17 (1.17)	4.03 (0.97)	0.25
Friend play with	4.25 (0.94)	4.05 (1.14)	0.10
Friend encourage	2.94 (1.33)	2.82 (1.28)	0.43
**Environmental**			
Safety	2.67 (0.51)	2.57 (0.53)	0.087
Walkability	2.99 (0.37)	2.93 (0.41)	0.20
Friendly neighbours	6.78 (6.71)	6.98 (7.38)	0.80
Clubs	3.82 (1.06)	3.69 (0.98)	0.24
Cost #	2.81 (0.63)	2.73 (0.68)	0.25
Playgrounds	1.05 (0.23)	1.03 (0.18)	0.36
Facilities	1.10 (0.29)	1.13 (0.33)	0.36

# reverse coded; Data presented as mean (standard deviation) unless specified otherwise; PAQ: Physical Activity Questionnaire; PA: Physical Activity.

**Table 2:** Unadjusted correlations among outcome and predictor variables.

Measured Variables	Boys			Girls		
	Daily Steps	PAQ Score	Sport	Daily Steps	PAQ Score	Sport
**Demographic**						
Education	−0.04	0.07	0.08	−0.04	−0.06	0.02
Income	−0.12	0.10	0.01	0.08	0.01	0.14 *
SEIFA score	−0.12 **	0.07	0.26 ***	0.05	−0.02	0.23 ***
**Personal**						
Age	−0.08	−0.02	0.01	−0.10 **	−0.11 ***	0.07
Screen time	0.08	0.14 ***	0.08	0.08	0.14 ***	0.006
Outcome expectancies	0.11 *	0.33 ****	0.35 ****	0.10 **	0.28 ****	0.27 ****
Barriers self-efficacy	0.13 **	0.30 ****	0.12	0.17 ****	0.35 ****	0.23 ***
Like PA	0.17 ***	0.38 ****	0.25 ***	0.25 ****	0.37 ****	0.27 ****
Good at PA	0.16 ***	0.36 ****	0.26 ***	0.27 ****	0.39 ****	0.31 ****
Don’t like PA feel #	0.05	0.18 ****	0.25 **	0.04	0.14 ****	0.13
**Interpersonal**						
Parent support	0.06	0.37 ****	0.43 ****	0.13 ***	0.38 ****	0.22 ***
Parent play with	0.07	0.33 ****	0.23 **	0.06	0.34 ****	0.09
Clothing	0.14 ***	0.30 ****	0.51 ****	0.08 *	0.30 ****	0.37 ****
Rules	0.08	0.14 ***	0.001	0.04	0.16 ****	0.10
Bullies #	0.01	0.17 ****	0.08	0.04	0.08 *	0.22 ***
Other kids tease #	0.02	0.01	0.05	0.05	0.02	0.12
Friend play with	0.17 ***	0.28 ****	0.06	0.07	0.38 ****	0.06 **
Friend encourage	0.23 ****	0.35 ****	0.14	0.11 **	0.39 ****	0.06
**Environmental**						
Safety	0.08	0.10	0.20 *	−0.01	0.07	0.07
Walkability	0.15 *	−0.09	0.12	0.15 **	0.05	0.16 *
Friendly neighbours	0.02	0.11	0.27 ***	0.05	0.10	0.15 *
Clubs	0.09	0.20 ****	0.30 ***	0.07	0.22 ****	0.33 ****
Cost #	−0.08	0.07	0.09	−0.04	−0.02	0.21 **
Playgrounds	−0.03	−0.18 **	−0.10	−0.02	−0.04	−0.04
Facilities	−0.09	−0.007	−0.20*	−0.01	0.04	−0.09

# reverse coded; * *p* < 0.05, ** *p* ≤ 0.01; *** *p* ≤ 0.001; **** *p* ≤ 0.0001.

**Table 3:** Regression Models of physical activity outcomes in boys.

Outcome Variables	Predictor Variables	Coefficient (Odds Ratio)	95% Confidence Interval	Robust SE	*p*
**Personal**					
Daily steps (*n* = 245)	No significant predictors				
PAQ score (*n* = 240)	Like PA	0.34	0.17–0.50	0.08	<0.0001
	Screen time	0.10	0.05–0.15	0.02	<0.0001
	Good at PA	0.19	0.07–0.32	0.06	0.004
	Barriers self-efficacy	0.14	0.02–0.25	0.06	0.023
		R^2^ = 0.29			
Sport (*n* = 236)	Like PA	2.24	1.26–4.01	0.67	0.006
	Outcome expectancies	2.54	1.31–4.93	0.86	0.006
	Don’t like PA feel #	0.14	1.02–2.14	0.06	0.033
		R^2^ = 0.19			
**Interpersonal**					
Daily steps (*n* = 245)	Clothing	701.70	116.7–1286.80	284.60	0.021
		R^2^ = 0.05			
PAQ score (*n* = 211)	Parent support	0.25	0.17–0.34	0.04	<0.0001
	Friend encourage	0.10	0.04–0.16	0.03	0.002
	Friend play with	0.13	0.04–0.21	0.04	0.005
		R^2^ = 0.27			
Sport (*n* = 124)	Parent support	2.70	1.42–5.14	0.89	0.002
	Clothing	2.40	1.44–4.00	0.62	0.001
		R^2^ = 0.22			
**Environmental**					
Daily steps (*n* = 140)	Walkability	1121.10	87.1–2155.20	503.10	0.03
		R^2^ = 0.02			
PAQ score (*n* = 145)	Clubs	0.25	0.02–0.30	0.04	<0.0001
		R^2^ = 0.02			
	Playgrounds	−0.46	−0.84–−0.08	0.19	0.02
		R^2^ = 0.09			
Sport (*n* = 130)	Clubs	1.93	1.23–3.03	0.44	0.004
		R^2^ = 0.08			

# reverse coded; All models controlled for sociodemographic variables (parent education, equivalised income and SEIFA score).

**Table 4:** Regression Models of physical activity outcomes in girls.

Outcome Variables	Predictor Variables	Coefficient (Odds Ratio)	95% Confidence Interval	Robust SE	*p*
**Personal**					
Daily steps (*n* = 242)	Good at PA	760.50	394.90–1126.10	176.70	<0.0001
	Barriers self-efficacy	516.00	61.20–970.90	219.90	0.03
	Like PA	926.70	529.90–1323.50	191.80	<0.0001
		R^2^ =			
		0.11 (with ‘Like PA’)			
		0.07 (with ‘Good at PA’)			
PAQ score (*n* = 295)	Good at PA	0.19	0.11–0.28	0.04	<0.0001
	Like PA	0.17	0.07–0.28	0.05	0.002
	Barriers self-efficacy	0.14	0.03–0.25	0.05	0.01
		R^2^ = 0.23			
Sport (*n* = 294)	Outcome expectancies	1.99	1.05–3.75	0.64	0.03
		R^2^ = 0.11			
**Interpersonal**					
Daily steps (*n* = 418)	Parent support	374.60	91.70–657.40	137.60	0.01
		R^2^ = 0.02			
PAQ score (*n* = 267)	Friend play with	0.19	0.11–0.26	0.05	<0.0001
	Clothing	0.17	0.07–0.27	0.06	0.002
	Parent support	0.13	0.01–0.24	0.06	0.04
		R^2^ = 0.32			
Sport (*n* = 269)	Clothing	2.15	1.24–3.74	0.61	0.006
	Parent support	2.10	1.15–3.85	0.65	0.016
		R^2^ = 0.23			
**Environmental**					
Daily steps (*n* = 238)	Walkability	856.10	118.30–1593.80	358.20	0.03
		R^2^ = 0.02			
PAQ score (*n* = 302)	Clubs	0.15	0.08–0.22	0.03	<0.0001
		R^2^ = 0.05			
Sport (*n* = 184)	No significant predictors				

All models controlled for sociodemographic variables (parent education, equivalised income and SEIFA score).

## References

[1] Poitras V.J., Gray C.E., Borghese M.M., Carson V., Chaput J.-P., Janssen I., Katzmarzyk P.T., Pate R.R., Connor Gorber S., Kho M.E. (2016). Systematic review of the relationships between objectively measured physical activity and health indicators in school-aged children and youth. Appl. Physiol. Nutr. Metab..

[2] Van Sluijs E.M.F., McMinn A.M., Griffin S.J. (2007). Effectiveness of interventions to promote physical activity in children and adolescents: Systematic review of controlled trials. BMJ.

[3] Dumith S.C., Gigante D.P., Domingues M.R., Kohl H.W. (2011). Physical activity change during adolescence: A systematic review and a pooled analysis. Int. J. Epidemiol..

[4] Telama R. (2009). Tracking of physical activity from childhood to adulthood: A review. Obes. Facts.

[5] Heitzler C.D., Martin S.L., Duke J., Huhman M. (2006). Correlates of physical activity in a national sample of children aged 9–13 years. Prev. Med..

[6] Sallis J., Owen N., Fisher E., Glanz K., Rimer B., Viswanath K., Orleans C. (2008). Ecological models of health behaviour. Health Behaviour and Health Education: Theory, Research, and Practice.

[7] Atkin A.J., van Sluijs E.M.F., Dollman J., Taylor W.C., Stanley R.M. (2016). Identifying correlates and determinants of physical activity in youth: How can we advance the field?. Prev. Med..

[8] Sterdt E., Liersch S., Walter U. (2014). Correlates of physical activity of children and adolescents: A systematic review of reviews. Health Educ. J..

[9] Lawman H.G., Wilson D.K., Van Horn M.L., Resnicow K., Kitzman-Ulrich H. (2011). The relationship between psychosocial correlates and physical activity in underserved adolescent boys and girls in the act trial. J. Phys. Act. Health.

[10] Stanley R.M., Ridley K., Dollman J. (2012). Correlates of children’s time-specific physical activity: A review of the literature. Int. J. Behav. Nutr. Phys. Act..

[11] Hanson M.D., Chen E. (2007). Socioeconomic status and health behaviors in adolescence: A review of the literature. J. Behav. Med..

[12] Wenthe P.J., Janz K.F., Levy S.M. (2009). Gender similarities and differences in factors associated with adolescent moderate-vigorous physical activity. Pediatr. Exerc. Sci..

[13] Stanley R.M., Ridley K., Olds T.S., Dollman J. (2014). Increasing specificity of correlate research: Exploring correlates of children’s lunchtime and after-school physical activity. PLoS ONE.

[14] Lubans D.R., Hesketh K., Cliff D.P., Barnett L.M., Salmon J., Dollman J., Morgan P.J., Hills A.P., Hardy L.L. (2011). A systematic review of the validity and reliability of sedentary behaviour measures used with children and adolescents. Obes. Rev..

[15] Troiano R.P., Berrigan D., Dodd K.W., Masse L.C., Tilert T., McDowell M. (2008). Physical activity in the United States measured by accelerometer. Med. Sci. Sports Exerc..

[16] Sirard J.R., Pate R.R. (2001). Physical activity assessment in children and adolescents. Sports Med..

[17] Ryan R.M., Patrick H., Deci E.L., Williams G.C. (2008). Facilitating health behaviour change and its maintenance: Interventions based on self-determination theory. Eur. Health Psychol..

[18] Zarnowiecki D., Ball K., Parletta N., Dollman J. (2014). Describing socioeconomic gradients in children’s diets—Does the socioeconomic indicator used matter?. Int. J. Behav. Nutr. Phys. Act..

[19] Zarnowiecki D.M., Parletta N., Dollman J. (2016). Socio-economic position as a moderator of 9–13-year-old children’s non-core food intake. Public Health Nutr..

[20] Australian Bureau of Statistics Census of Population and Housing (2008). Socio-Economic Indexes for Areas (Seifa).

[21] McMinn D., Rowe D., Stark M., Nicol L. (2010). Validity of the new lifestyles NL1000 accelerometer for measuring time spent in moderate-to-vigorous physical activity in school settings. Meas. Phys. Educ. Exerc. Sci..

[22] Liggett L., Gray A., Parnell W., McGee R., McKenzie Y. (2012). Validation and reliability of the new lifestyles NL-1000 accelerometer in New Zealand preschoolers. J. Phys. Act. Health.

[23] McClain J.J., Hart T.L., Getz R.S., Tudor-Locke C. (2010). Convergent validity of 3 low cost motion sensors with the actigraph accelerometer. J. Phys. Act. Health.

[24] Rowe D.A., Mahar M.T., Raedeke T.D., Lore J. (2004). Measuring physical activity in children with pedometers: Reliability, reactivity, and replacement of missing data. Pediatr. Exerc. Sci..

[25] Janz K.F., Lutuchy E.M., Wenthe P., Levy S.M. (2008). Measuring activity in children and adolescents using self-report: Paq-c and Paq-a. Med. Sci. Sports Exerc..

[26] Kowalski K.C., Crocker P.R.E., Kowalski N.P. (1997). Convergent validity of the physical activity questionnaire for adolescents. Pediatr. Exerc. Sci..

[27] Pettman T., McAllister M., Verity F., Margarey A., Dollman J., Triptree M., Stanley S., Wilson A., Mastersson N. (2010). Eat Well Be Active Community Programs Final Report.

[28] Ball K., Timperio A., Salmon J., Giles-Corti B., Roberts R., Crawford D. (2007). Personal, social and environmental determinants of educational inequalities in walking: A multilevel study. J. Epidemiol. Community Health.

[29] Ball K., Cleland V.J., Timperio A.F., Salmon J., Giles-Corti B., Crawford D.A. (2010). Love thy neighbour? Associations of social capital and crime with physical activity amongst women. Soc. Sci. Med..

[30] Australian Bureau of Statistics (2000). Measuring Social Capital: Current Collections and Future Directions.

[31] Hallal P.C., Andersen L.B., Bull F., Guthold R., Haskell W., Ekelund U. (2012). Global physical activity levels: Surveillance progress, pitfalls, and prospects. Lancet.

[32] Pearce M.S., Basterfield L., Mann K.D., Parkinson K.N., Adamson A.J., Reilly J.J. (2012). Early predictors of objectively measured physical activity and sedentary behaviour in 8–10 years old children: The gateshead millennium study. PLoS ONE.

[33] Van Der Horst K., Paw M.J., Twisk J.W., Van Mechelen W. (2007). A brief review on correlates of physical activity and sedentariness in youth. Med. Sci. Sports Exerc..

[34] Stokols D. (1992). Establishing and maintaining healthy environments. Toward a social ecology of health promotion. Am. Psychol..

[35] Spence J.C., Lee R.E. (2003). Toward a comprehensive model of physical activity. Psychol. Sport Exerc..

[36] Telford R.M., Telford R.D., Olive L.S., Cochrane T., Davey R. (2016). Why are girls less physically active than boys? Findings from the look longitudinal study. PLoS ONE.

[37] Seabra A., Mendonca D., Maia J., Welk G., Brustad R., Fonseca A.M., Seabra A.F. (2013). Gender, weight status and socioeconomic differences in psychosocial correlates of physical activity in schoolchildren. J. Sci. Med. Sport.

[38] Eccles J., Wigfield A., Harold R.D., Blumenfeld P. (1993). Age and gender differences in children’s self- and task perceptions during elementary school. Child Dev..

[39] Pellegrini A.D., Blatchford P., Kato K., Baines E. (2004). A short-term longitudinal study of children’s playground games in primary school: Implications for adjustment to school and social adjustment in the USA and the UK. Soc. Dev..

[40] Keay J. (2007). Learning from other teachers: Gender influences. Eur. Phys. Educ. Rev..

[41] Cox A.E., Smith A.L., Williams L. (2008). Change in physical education motivation and physical activity behavior during middle school. J. Adolesc. Health.

[42] DiLorenzo T.M., Stucky-Ropp R.C., Vander Wal J.S., Gotham H.J. (1998). Determinants of exercise among children. II. A longitudinal analysis. Prev. Med..

[43] Allender S., Cowburn G., Foster C. (2006). Understanding participation in sport and physical activity among children and adults: A review of qualitative studies. Health Educ. Res..

[44] Michael S.L., Coffield E., Lee S.M., Fulton J.E. (2016). Variety, enjoyment, and physical activity participation among high school students. J. Phys. Act. Health.

[45] Liu J., Sun H., Beets M.W., Probst J.C. (2013). Assessing natural groupings of common leisure-time physical activities and its correlates among U.S. adolescents. J. Phys. Act. Health.

[46] Chiarlitti N.A., Kolen A.M. (2017). Parental influences and the relationship to their children’s physical activity levels. Int. J. Exerc. Sci..

[47] Ferreira I., van der Horst K., Wendel-Vos W., Kremers S., van Lenthe F.J., Brug J. (2007). Environmental correlates of physical activity in youth—A review and update. Obes. Rev..

[48] Ding D., Sallis J.F., Kerr J., Lee S., Rosenberg D.E. (2011). Neighborhood environment and physical activity among youth. Am. J. Prev. Med..

[49] Booth V., Walsh E., Dollman J. (2017). The demographic influence on physical activity trends among South Australian children and adolescents. J. Sci. Med. Sport.

[50] Marques A., Ekelund U., Sardinha L.B. (2016). Associations between organized sports participation and objectively measured physical activity, sedentary time and weight status in youth. J. Sci. Med. Sport.

[51] Australian Bureau of Statistics (2012). Children’s Participation in Sport and Leisure Time Activities, 2003–2012.

